# Penile bacteria associated with HIV seroconversion, inflammation, and immune cells

**DOI:** 10.1172/jci.insight.147363

**Published:** 2021-04-22

**Authors:** Jessica L. Prodger, Alison G. Abraham, Aaron A.R. Tobian, Daniel E. Park, Maliha Aziz, Kelsey Roach, Ronald H. Gray, Lane Buchanan, Godfrey Kigozi, Ronald M. Galiwango, Joseph Ssekasanvu, James Nnamutete, Joseph Kagaayi, Rupert Kaul, Cindy M. Liu

**Affiliations:** 1 Department of Microbiology and Immunology and; 2 Department of Epidemiology and Biostatistics, Schulich School of Medicine and Dentistry, Western University, London, Ontario, Canada.; 3 Department of Epidemiology, School of Public Health, and; 4 Department of Ophthalmology, School of Medicine, University of Colorado Denver, Denver, Colorado, USA.; 5 Department of Epidemiology, Bloomberg School of Public Health, and; 6 Department of Pathology, Johns Hopkins University School of Medicine, Johns Hopkins University, Baltimore, Maryland, USA.; 7 Department of Environmental and Occupational Health, Milken Institute School of Public Health, George Washington University, Washington, DC, USA.; 8 Rakai Health Sciences Program, Kalisizo, Uganda.; 9 Department of Medicine and; 10 Department of Immunology, University of Toronto, Toronto, Ontario, Canada.; 11 Division of Infectious Diseases, University Health Network, Toronto, Ontario, Canada.

**Keywords:** AIDS/HIV, Bacterial infections, Cytokines, T cells

## Abstract

The foreskin is a site of heterosexual acquisition of HIV-1 among uncircumcised men. However, some men remain HIV-negative despite repeated, unprotected vaginal intercourse with HIV-positive partners, while others become infected after few exposures. The foreskin microbiome includes a diverse group of anaerobic bacteria that have been linked to HIV acquisition. However, these anaerobes tend to coassociate, making it difficult to determine which species might increase HIV risk and which may be innocent bystanders. Here, we show that 6 specific anaerobic bacterial species, *Peptostreptococcus anaerobius*, *Prevotella bivia*, *Prevotella disiens*, *Dialister propionicifaciens*, *Dialister micraerophilus*, and a genetic near neighbor of *Dialister succinatiphilus*, significantly increased cytokine production, recruited HIV-susceptible CD4^+^ T cells to the inner foreskin, and were associated with HIV acquisition. This strongly suggests that the penile microbiome increases host susceptibility to HIV and that these species are potential targets for microbiome-based prevention strategies.

## Introduction

Approximately 1.7 million people are newly infected by HIV-1 each year (1). The composition of the bacterial community on the foreskin modifies a heterosexual man’s risk for HIV acquisition (2), as is the case for the vaginal bacterial community in a woman (3, 4), and may contribute to the well-established protective effect of male circumcision (5–8). However, the bacterial species responsible for increased HIV risk and the biological mechanism by which they facilitate HIV acquisition are unclear. Closing these knowledge gaps may open doors for novel HIV prevention strategies.

The foreskin mucosa is a complex microenvironment in which rich communities of immune cells and microbes interact. The immune milieu incorporates T and B cells, dendritic cells, and innate immune cells, along with their associated cytokines and antimicrobial peptides, and protects against HIV acquisition after most sexual exposures (9). However, microbes can activate immune cells, making them more susceptible to HIV; therefore, the bacterial species that comprise the foreskin microbiome may be a key determinant of HIV susceptibility. We present for the first time, to our knowledge, a comprehensive analysis of the penile bacterial microbiome in 2 previously described, independent cohorts of HIV-uninfected and uncircumcised men from rural Uganda (6, 10) in order to identify the exact foreskin bacterial species associated with enhanced HIV acquisition and to determine whether these species drive HIV acquisition by recruiting HIV-susceptible CD4^+^ T cells to vulnerable regions of the inner foreskin.

## Results

### Penile bacterial communities associated with HIV acquisition.

We initially performed a comprehensive assessment of foreskin bacterial communities from HIV-uninfected men, using coronal sulcus swabs collected from men in the uncircumcised control arm of a randomized trial of male circumcision for HIV prevention in Rakai, Uganda (6). We performed a case-control study to estimate the ORs of subsequent HIV seroconversion associated with specific bacterial species present at baseline. For this HIV seroconversion cohort (6), cases (*n =* 48) were defined as uncircumcised men who went on to acquire HIV, and controls (*n =* 140) were uncircumcised men who remained HIV-uninfected during the full 24-month period of follow-up.

Total bacterial density was highly variable among the 188 study participants, spanning 5 orders of magnitude. Several bacterial genera were prevalent in all men, including *Prevotella*, *Peptoniphilus*, *Porphyromonas*, *Finegoldia*, *Corynebacterium*, *Anaerococcus*, *Dialister*, and a genetic near-neighbor of the family *Peptoniphilaceae*. Although prevalent in all men, the abundances of these genera within the penile microbiome varied substantially among men. *Prevotella* was the most abundant, with a median proportional abundance of 15.5% (IQR, 5.9%–27.9%; Supplemental Table 1; supplemental material available online with this article; https://doi.org/10.1172/jci.insight.147363DS1).

**Figure 1:**
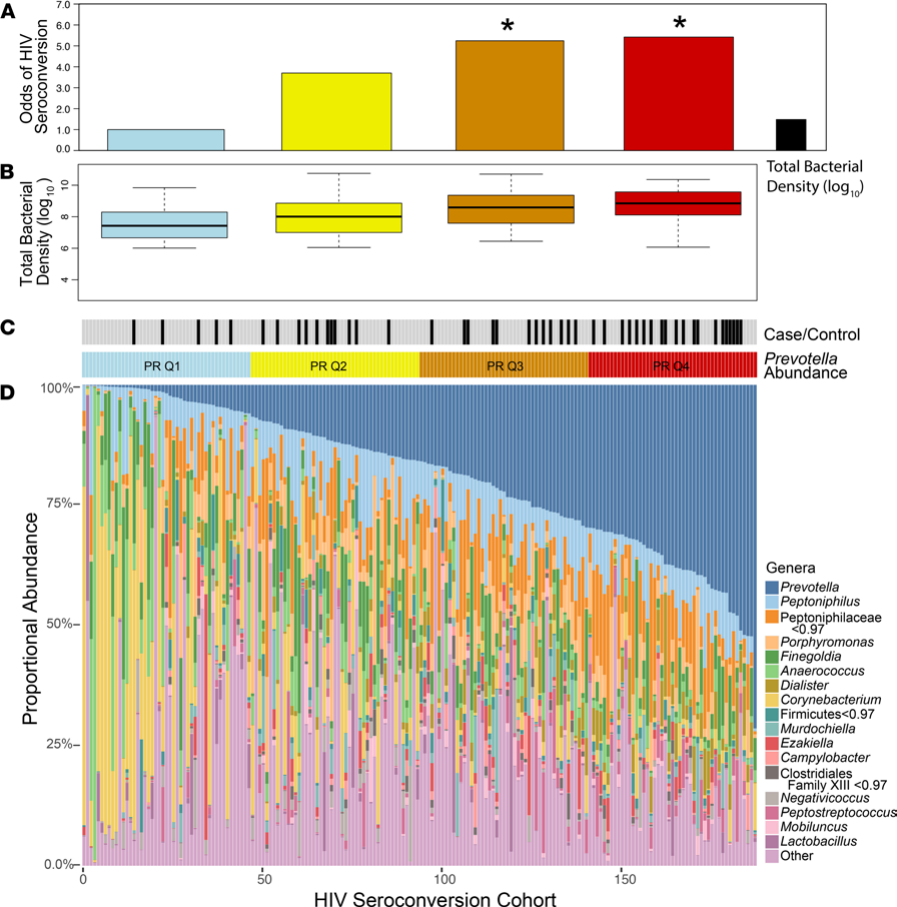
Men in the 2 highest quartiles of *Prevotella* abundance are at significantly higher risk for subsequent HIV seroconversion. *Prevotella* proportional abundance (**A**) and total bacterial load (**B**) are associated with increased odds of HIV seroconversion. Case/control status and *Prevotella* quartile assignments are shown in **C**. Proportional abundances of penile bacterial genera for all men in the HIV seroconversion cohort (*n =* 188) are shown as a stacked bar plot in **D**. Logistic regression analyses adjusted for number of sexual partners, condom use, and age. *P < 0.05. PR Q, *Prevotella* quartile.

To identify the bacterial communities associated with HIV acquisition, we first examined the overall penile microbiome composition based on the proportional abundance of *Prevotella* (Figure 1). Men in the 2 highest *Prevotella* quartiles had a significantly higher risk of seroconversion compared with those in the lowest quartile (Figure 1A; Q3, aOR = 5.24, 95%CI = 1.43–22.60; Q4, aOR = 5.42, 95%CI = 1.63–21.19; adjusting for age, number of sex partners, and condom use). Total bacterial density differed significantly across the 4 quartiles (Figure 1B; ANOVA, *P <* 0.001) and was also significantly (but not as strongly) associated with risk of seroconversion (Figure 1A; OR = 1.34, 95% CI = 1.01–1.82).

### Defining specific bacterial species uniquely associated with HIV acquisition and inflammation.

Because several genera frequently co-occurred with *Prevotella*, we performed a nontargeted search within the HIV seroconversion cohort sample set to differentiate HIV seroconversion-associated species from bystanders. Eleven penile bacterial genera were significantly associated with increased risk of seroconversion (Supplemental Table 2). We extracted and analyzed species-level data from these 11 seroconversion-associated genera as well as from an additional 6 genera previously associated with HIV risk (Supplemental Table 3). Of the 56 species belonging to these 17 genera, 34 were positively associated with seroconversion (aOR, *P <* 0.05; Table 1). No bacterial species were associated with decreased risk of seroconversion (i.e., with protection against HIV acquisition).

To understand the mechanisms by which these species might increase risk, we first used subpreputial swabs collected from the HIV seroconversion cohort to define bacterial associations with foreskin soluble immune parameters previously associated with increased HIV risk, specifically IL-8 and α-defensins (11–16). All 34 bacterial species were associated with elevated subpreputial IL-8 levels, and 94.1% (32 of 34 species) were associated with elevated α-defensins (Supplemental Table 4). To assess additional correlations with tissue immune cells that represent preferential targets for HIV infection, we then performed for the first time to our knowledge an analysis of the penile bacterial microbiome in a second cohort of HIV-uninfected, uncircumcised men. This independent cellular immunology cohort, for which both subpreputial swabs and foreskin tissues had been collected, comprised a cross-sectional study of HIV-negative Ugandan men undergoing elective circumcision for HIV prevention between 2009 and 2012 (17). In this separate cohort, most seroconversion-associated bacterial species were again associated with both elevated subpreputial IL-8 (32 of 34 species) and elevated α-defensins levels (27 of 34 species; Supplemental Table 4). However, only 6 species (17.6%) were additionally correlated with an increased foreskin tissue density of preferential HIV target cells (CCR5^+^CD4^+^ T cells; Supplemental Table 4). These 6 species were *Peptostreptococcus anaerobius*, *Prevotella bivia*, *Prevotella disiens,* *Dialister propionicifaciens, Dialister micraerophilus*, and a genetic near neighbor of *Dialister succinatiphilus* (Table 2). We named these species “bacteria associated with HIV seroconversion, inflammation, and immune Cells” (BASIC). These BASIC species are strict anaerobes, and each was associated with increased subpreputial proinflammatory immune mediators (IL-8 and α-defensins), density of foreskin HIV target cells (CCR5^+^CD4^+^ T cells and Th17 cells; Supplemental Table 4), and HIV seroconversion.

### Distinguishing between the effects of total bacterial density and BASIC species.

**Figure 2:**
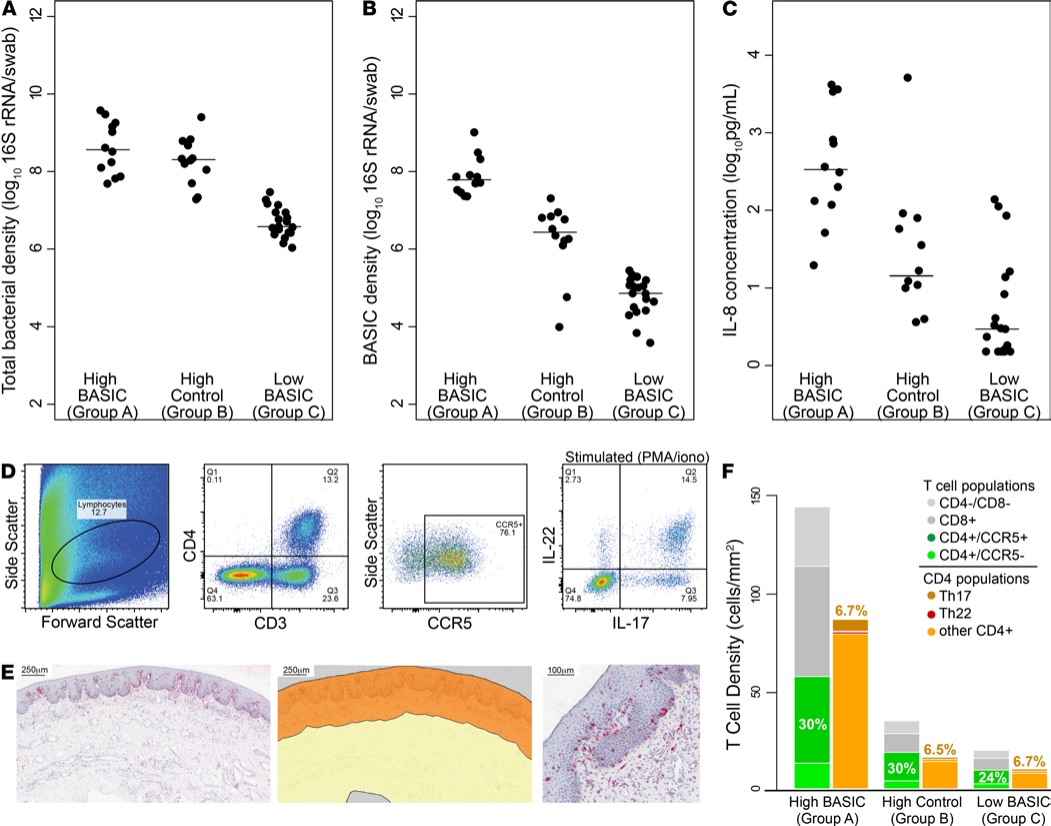
High abundance of BASIC species, but not overall bacterial load, is associated with high foreskin T cell density without alterations in T cell subset distribution. Men in the cellular immunology cohort were divided into 3 groups, high BASIC species (group A, *n =* 12), high control taxa (group B, *n =* 12), and low BASIC species (group C, *n =* 19), and total bacterial density (**A**), BASIC species density (**B**), and subpreputial IL-8 (**C**), were compared. Foreskin T cell immune response was measured using flow cytometry (**D**) combined with CD3 immunohistochemistry (**E**) (analysis area highlighted in orange; scale bar: 250 μm [left and middle]; 200 μm [right]), and showed that group A had significantly (both *P* < 0.05) higher foreskin T cell density than groups B or C (**F**). Numbers in **F** represent percentages of CD3^+^ cells that are CD4^+^CCR5^+^ and Th17 cells (Wilcoxon rank-sum test).

To distinguish between the foreskin inflammatory effects of an increased overall bacterial density and those specific to an increased density of BASIC species, we selected 4 control bacterial taxa as a comparator to the BASIC species: these included 2 taxa of anaerobes reduced by male circumcision but not associated with seroconversion (*Negativicoccus spp*. and *Helcococcus spp.*) and 2 taxa that increased after male circumcision (*Corynebacterium spp*. and *Staphylococcus spp.*). We then categorized participants in the cellular immunology cohort into 3 groups: group A, in the top quartile of abundance for BASIC species but not for control taxa (*n =* 12); group B, in the top quartile of abundance for control taxa but not for BASIC species (*n =* 12); and group C, in the bottom quartile of BASIC species abundance (*n =* 19). No men met the criteria for more than 1 group. Groups A and B had similarly high median total penile bacterial densities (Figure 2, A and B; 3.67 × 10^8^ vs. 2.03 × 10^8^ 16S rRNA copies/swab, *P =* 0.30), but group A had a significantly higher density of BASIC species compared with group B (6.2 × 10^7^ vs. 2.8 × 10^6^ 16S rRNA copies/swab, *P <* 0.001). Group C was low in both total bacterial density and BASIC species density (Figure 2, A and B; *P <* 0.001 vs. groups A and B). Despite similarity in total bacterial densities, men in group A had much higher subpreputial IL-8 levels than men in group B (336.1 vs. 14.4 pg/ml, *P =* 0.001), while men in group C had the lowest levels of IL-8 (3.0 pg/ml, *P <* 0.01 vs. groups A and B; Figure 2C), suggesting that the BASIC species, above and beyond any immune effects of total bacterial load, were potent inducers of penile IL-8.

We found that T cell recruitment to the foreskin tissues was also strongly associated with BASIC species (Figure 2, D–F). The relative abundances of foreskin T cell subsets were quantified using flow cytometry (Figure 2D), and the overall tissue density of T cells was quantified using immunohistochemistry (Figure 2E). The median density of HIV target cells in the foreskin tissue, including CCR5^+^CD4^+^ T cells and highly HIV-susceptible Th17 cells (15), was nearly 6-fold higher among men in group A, as compared with men in group B or C (30.0 vs. 6.2 and 4.0 CCR5^+^CD4^+^ T cells/mm^2^; 6.1 vs. 1.2 and 0.6 Th17 cells/mm^2^; all *P <* 0.05; Figure 2F and Supplemental Table 5). However, the relative proportions of specific T cell subsets were similar across all 3 groups, suggesting that BASIC bacteria were not inducing the recruitment of specific T cell subsets to the foreskin tissue (Figure 2F and Supplemental Table 6). Taken together, these data suggest that BASIC species may increase HIV seroconversion risk through the generalized tissue recruitment of T cells to the foreskin, including highly HIV-susceptible target cells.

### Tissue compartmentalization of inflammation induced by BASIC species.

**Figure 3:**
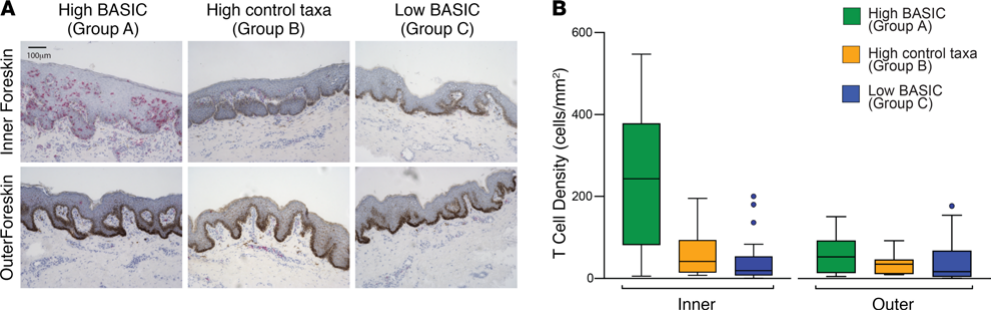
High BASIC species abundance is associated with T cell recruitment to only the inner foreskin. As shown by representative CD3 histology images (scale bar: 100 μm) (**A**), men with high abundance of BASIC species (group A, *n =* 12) had significantly (both *P* < 0.01) higher inner foreskin T cell densities than groups B (*n =* 12) and C (*n =* 19) (**B**). The increased T cell abundance was restricted to the inner aspect of the foreskin (which is exposed to subpreputial anaerobes); outer foreskin T cell densities were similarly low in all 3 groups (Wilcoxon rank-sum test).

The foreskin forms a fold of skin over the glans on the nonerect penis and thus comprises two aspects: the outer foreskin, which is exposed to the air on the nonerect penis, and the inner foreskin, which lies against the glans on the nonerect penis and is exposed to the comparatively hypoxic subpreputial space and coronal sulcus bacteria. We compared T cell density between the inner and outer foreskin aspects and found that T cells were specifically localized in the inner foreskin in men with high BASIC species (Figure 3). Men in group A had over 5-fold higher median T cell density in the inner foreskin than men in groups B and C (243.1 vs. 41.5 and 19.2 T cells/mm^2^, both *P <* 0.01). However, in the outer foreskin, median T cell densities were low in all 3 groups (53.8, 35.8, and 17.2 T cells/mm^2^) with no significant differences between groups (Figure 3B). These findings are consistent with T cell recruitment occurring in response to the anaerobic BASIC species in the low-oxygen subpreputial space of the inner foreskin.

### Penile bacterial community network patterns.

**Figure 4:**
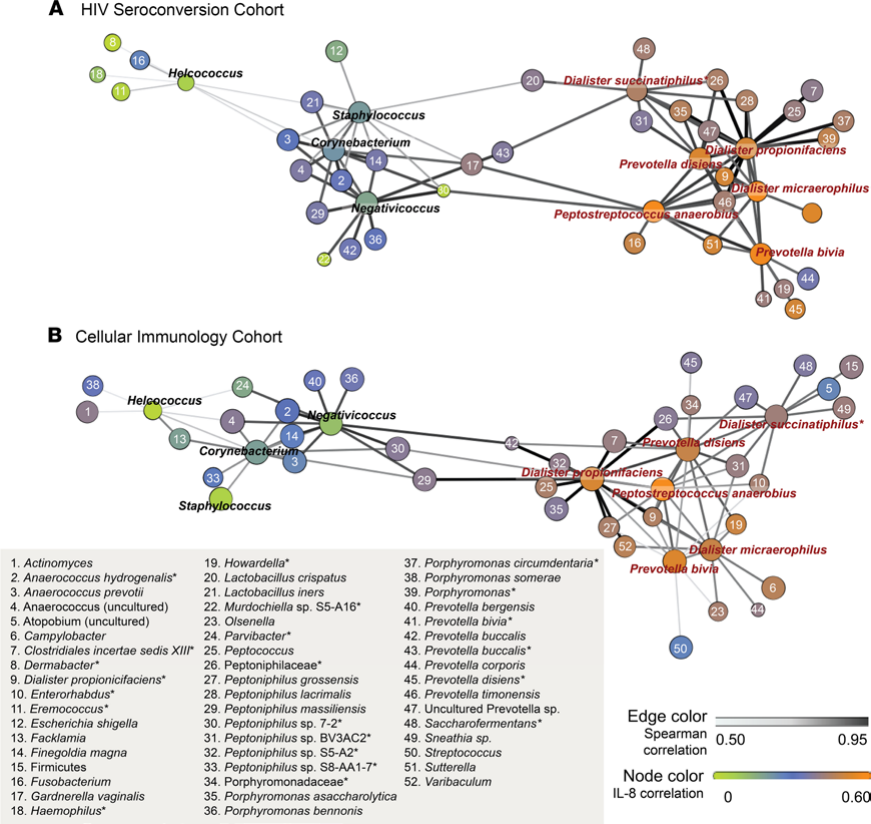
Penile bacterial community network structure is conserved across cohorts, with clustering of BASIC species. Penile microbiome network structures are conserved across 2 independent study cohorts, the HIV seroconversion cohort (*n* = 188) (**A**) and the cellular immunology cohort (*n* = 90) (**B**). Network structure displays the correlation of the 6 BASIC species and their 10 most correlated genera/species (species are listed). Correlation between species is denoted by the spatial location of nodes and edge color; node size displays species prevalence. Species-level correlations with subpreputial IL-8 are displayed by node color. Correlations were assessed by Spearman’s test.

**Figure 5:**
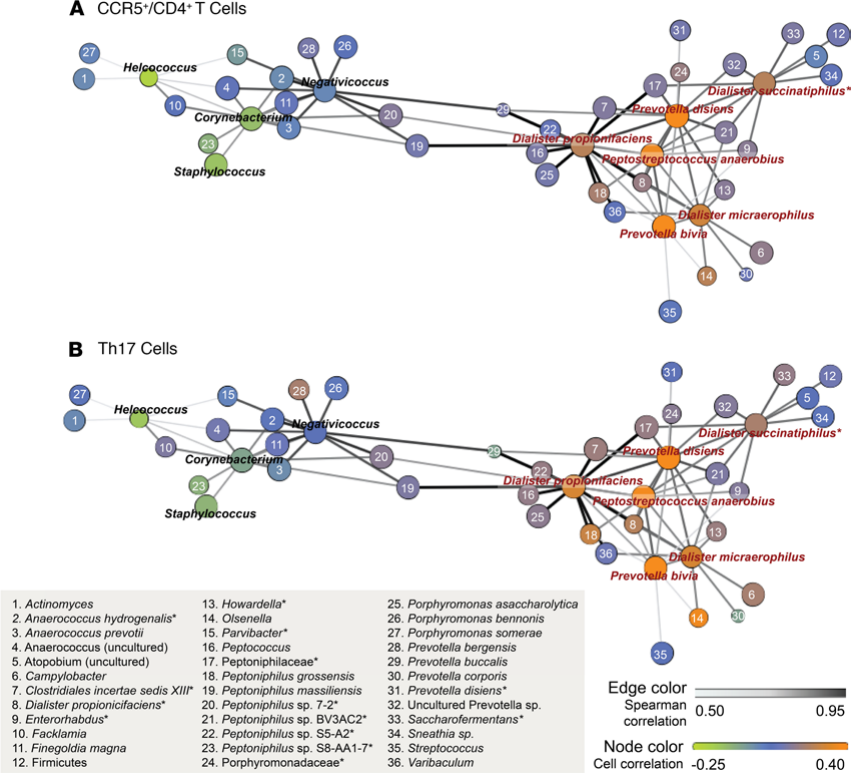
Penile microbiome networks identify a highly co-occurring triad that is strongly associated with HIV target cell density in the cellular immunology cohort (*n* = 90). Node colors indicate species-level associations with HIV target cell density, including CCR5^+^CD4^+^ T cells (**A**) and Th17 cells (**B**). Correlation between species denoted by spatial location of nodes and edge color and node size displays species prevalence. Correlations were assessed by Spearman’s test.

Finally, we assessed the co-occurrence patterns of the BASIC species to determine whether they occurred in specific bacterial networks. Using data from the HIV seroconversion cohort, network analysis revealed that the BASIC species and the 4 control taxa formed 2 distinct network substructures (Figure 4A), and this bacterial network structure was strikingly similar to that of the independent cellular immunology cohort (Figure 4B). Additionally, 3 of the 6 BASIC species — *P*. *anaerobius*, *D*. *micraerophilus*, and *P*. *bivia* — formed a close triad, which was highly associated with HIV target cell density in the cellular immunology cohort, including CCR5^+^CD4^+^ T cells (Figure 5A) and Th17 cells (Figure 5B). *D*. *propionicifaciens* correlated highly with other bacteria in the BASIC substructure and served as a network hub (Figures 4 and 5). None of the bacteria that co-occurred with the BASIC species, nor the control taxa, were associated with HIV target cell density (Figure 5, Table 2, and Supplemental Table 6). Overall, this indicates that a few, highly co-occurring species were associated with host inflammatory immune parameters that increase seroconversion risk.

## Discussion

The foreskin is the major site of HIV acquisition in uncircumcised heterosexual men, and HIV risk is reduced by almost 60% after penile circumcision (6–8). The mechanism of this protection is incompletely understood, and a better understanding may lead to new avenues for HIV prevention for the large subset of men who prefer not to undergo this surgical procedure. In the current study, we identified 6 BASIC anaerobic bacterial species that are associated with each of elevated inflammatory immune mediators in the coronal sulcus, compartmentalized recruitment of HIV target cells to the inner foreskin, and increased HIV acquisition. This suggests that clinical interventions targeting these specific penile bacterial species should be explored in future HIV prevention studies.

Prior studies have shown that several anaerobic bacterial genera commonly found on the penis are reduced after circumcision (5) and that among men who remain uncircumcised most of these same genera are associated with foreskin inflammation and HIV acquisition (2). However, because anaerobic bacteria tend to coassociate, standard approaches to analyze seroconversion associations were not able to distinguish causation from coassociation. In the current study, we confirmed that the uncircumcised penile microbiome is largely comprised of highly co-occurring anaerobes, but a more detailed immune analysis and species-level taxonomic assignment demonstrated that only 6 species in the uncircumcised penile microbiome were associated with HIV seroconversion, proinflammatory cytokine levels, and foreskin tissue density of CD4^+^ T cells bearing the HIV coreceptor CCR5. While overall bacterial density was an important determinant of foreskin tissue inflammation, these key BASIC species were associated with much higher levels of tissue inflammation than control species, after controlling for bacterial density. Network analysis demonstrated that BASIC species clustered together, with *D*. *propionicifaciens* serving as a network hub for anaerobic bacteria. The particularly strong co-occurrence of the 3 BASIC species *P*. *anaerobius*, *D*. *micraerophilus*, and *P*. *bivia* is very interesting, given that, in women with bacterial vaginosis (BV), a commensal symbiosis has been described, wherein *P*. *bivia* supports the growth of *P*. *anaerobius* (18). Therefore, further mechanistic analyses may reveal specific targets to destabilize inflammatory penile anaerobe networks. This is the first biological evidence to our knowledge of a species-specific connection between penile bacteria and foreskin HIV-susceptible cell recruitment; this suggests targets for focused microbiome-specific interventions to reduce HIV transmission. Nonetheless, anaerobe co-occurrence makes it difficult to be certain of causation, and in vitro assays or perhaps in vivo animal models may be useful approaches to confirm their causal role.

Interestingly, no bacterial species or genera were found to be protective against penile HIV acquisition. *Lactobacillus crispatus*, a dominant component of the vaginal microbiome that is associated with reduced inflammation and relative HIV protection in women (3, 19), was associated with an increased risk of HIV acquisition in men and with increased penile IL-8 detection, although not with the density of HIV target cell subsets in foreskin tissue. While it is possible that the biological effect of *L*. *crispatus* is different from that of the anaerobes, it is also plausible that the detection of this species within the penile microbiome is a marker for recent unprotected sex with a female partner, where the genital environment is enriched for IL-8 as well as these bacteria (20), rather than representing a stable component of the penile microbiome with direct host immune impact.

Indeed, despite substantial overlap in key bacterial species and taxa, the structure of the penile microbiome of an uncircumcised man is quite distinct from that of the vagina. While there is no universally accepted classification system for the female genital tract microbiome, it can be fairly easily sorted into distinct community state types (CSTs) that are predominated either by one of several *Lactobacillus* species or by *Gardnerella vaginalis*, with or without diverse “BV-associated anaerobes,” and these CSTs are in turn associated with very distinct host mucosal immune profiles (19, 20). While one prior study was able to sort the penile microbiota into 7 CSTs (21), none of these were predominated by *Lactobacillus* species, and they were not nearly as distinct as those in the female genital tract. Rather, in this current, much larger analysis, we demonstrated a core repertoire of common penile taxa/species that can vary substantially from one person to another in terms of their relative abundance and confirm that lactobacilli do not play a central role.

The current work uses sophisticated microbiological, immunological, and statistical techniques to define the associations among penile bacteria, immunology, and HIV acquisition. Models that could better define the causal nature of these relationships might include small- or large-animal models, ex vivo explant models, or in vitro epithelial constructs. While such assays are beyond the scope of the current work, prior work from our group demonstrates that the associations we define are likely to be causal. Specifically, the clinical treatment of BV in women caused a reduction in *Prevotella* spp, reduced HIV entry into cervical CD4^+^ T cells, and dramatically changed genital cytokine levels, clearly demonstrating a causal relationship between female genital bacteria and genital immunology (22). Furthermore, the application of inflammatory cytokines to foreskin explant tissues recruited CD4^+^ T cell targets to the epithelium of the inner (but not outer) foreskin (23). In the latter case, these CD4^+^ T cells must have been mobilized from within the existing foreskin tissue, while in vivo one would expect even more substantive effects through broader tissue T cell recruitment in response to inflammation.

At present, it is not clear if or how these associations among the penile microbiome, local immunology, and HIV acquisition could be translated into clinical strategies to reduce HIV risk for uncircumcised men. It has been demonstrated in the Rakai community cohort that penile washing immediately after sex does not reduce HIV acquisition (and may actually increase it) (24). While the timing of washing in this study means that it could not have altered the penile microbiome at the time of sexual HIV exposure, even washing the penis immediately before sex would not be expected to immediately reverse the cellular immune associations of penile dysbiosis that we describe. Whether systemic or topical antibiotics can reduce the density of penile BASIC bacteria and/or alter penile immunology in uncircumcised men will be an interesting area for future research.

In conclusion, we have identified 6 bacterial species on the penis that were prospectively associated with HIV seroconversion and localized recruitment of the most susceptible HIV target cells to the inner foreskin. These BASIC species belong to genera that are reduced by male circumcision, which may explain why circumcised men are at reduced risk for HIV acquisition through heterosexual sex. The consistent cross-cohort association between BASIC species and penile inflammation strongly implicates the penile microbiome in host susceptibility to HIV infection and suggests that BASIC species may be candidates for novel microbiome-based prevention strategies.

## Methods

### Experimental design

The objective of this study was to identify subpreputial bacterial species associated with inflammation and increased risk of HIV seroconversion in uncircumcised heterosexual men. Two study designs were used to meet this objective: a case-control study using the HIV seroconversion cohort (6) and an observational, cross-sectional cohort study using the cellular immunology cohort (10). All samples were deidentified for analysis such that investigators performing assays were blinded to participant characteristics.

#### HIV seroconversion cohort.

We conducted a case-control study of uncircumcised adult men nested within a randomized trial of male circumcision for HIV prevention in 2004–2006 in Rakai, Uganda (6). As male circumcision significantly alters the penile microbiome (5), only trial participants from the delayed circumcision arm of the trial who remained uncircumcised for the duration of the trial were included. Each case was matched to 3 controls selected at random as long, as they had corresponding follow-up visits. Cases (*n =* 68) were men who acquired HIV during the 24-month follow-up, and controls (*n =* 140) were persistently HIV-uninfected men. We analyzed penile microbiome and subpreputial cytokines using coronal sulcus swabs collected at trial baseline.

#### Cellular immunology cohort.

We conducted a second study in a cohort of HIV-negative men (*n =* 89) presenting for elective adult male circumcision at the Rakai Circumcision Service Program between 2010 and 2011 (10, 17, 25). Participants with evidence of genital infections, such as urethral discharge, genital ulceration, or dysuria, were treated and reassessed to ensure resolution of infection before surgery. HIV-negative status was confirmed by HIV serology and real-time PCR. We analyzed penile microbiome and subpreputial cytokines using coronal sulcus swabs collected before surgical cleansing and the foreskin cellular immune response using foreskin tissues removed during surgery.

### Sample collection and processing

For both cohorts, clinical officers collected penile swabs using premoistened Dacron swabs and rotated twice around the full circumference of the penis at the coronal sulcus as previously described (6). DNA isolation for the HIV seroconversion cohort and the cellular immunology cohort was performed as described previously (21, 26).

To study foreskin cellular immunology, tissue removed during male circumcision was processed within 1 hour of surgery, as previously described (10). One section from each of the inner and outer aspects of the foreskin was snap frozen into cryomolds in OCT compound (Fisher Scientific) for immunohistochemistry; and one large section, containing equal area of the inner and outer aspects, was reserved for T cell isolation. The inner foreskin is the aspect of tissue that lies against the glans and coronal sulcus on the nonerect penis, while the outer foreskin is the aspect that is exposed to the air on the nonerect penis and is closer to the skin that remains on the shaft after circumcision.

### Penile microbiome characterization

Penile coronal sulcus microbiome analysis was characterized by 16S rRNA gene–based amplicon sequencing (V3V4) and by broad-range real-time PCR (V3V4) as previously described (21, 27). Additional species-level classification was performed using a custom Bayesian classifier trained by a curated training set created for select genera. Using the resultant sequencing and qPCR data, absolute abundance of each penile bacterial genus and species was calculated as follows: absolute abundance of a taxon per swab = total penile bacterial load per swab (measured as total copies of 16S rRNA gene per swab by qPCR) × proportional abundance of the given taxon (measured as the number of 16S rRNA gene sequences assigned to a taxon in a given sample, divided by the total number of 16S rRNA sequences obtained for the sample).

### Quantification of immune outcomes

Subpreputial IL-8 and a-defensins (human neutrophil peptides 1–3 [HNP1–HNP3]) were measured in coronal sulcus swabs, selected based on their previous association with HIV-1 acquisition in men and women (10–12). IL-8 was measured as part of a custom Human Ultra-Sensitive kit on an electrochemiluminescent detection system (Meso Scale Discovery; LLOQ for IL-8 = 1.5 pg/ml). HNP1–HNP3 were measured using conventional EIA (Hycult Biotechnology; LLOQ = 156 pg/ml).

T cells from tissues obtained through the cellular immunology cohort were characterized by a combination of flow cytometry and immunohistochemistry, as previously described (11, 25). T cells were isolated using a combination of mechanical and enzymatic disruption. Th17 and Th22 cells were identified functionally by the production of IL-17 or the production of IL-22 in the absence of IL-17, respectively, in response to stimulation with 1 ng/ml phorbol-12-myristate-13-acetate (MilliporeSigma) and 1 μg/ml ionomycin (MilliporeSigma) in the presence of 5 μg/ml Brefeldin A (GolgiPlug, BD Biosciences). Cells were stained with antibodies raised against CD3 (UCHT1), CD4^+^ (RPA-T4), CD8^+^ (SK1), CCR5 (2D7/CCR5; all BD Biosciences) and IL-17 (eBio64DEC17) and IL-22 (22URTI; both eBiosciences), and data were acquired using a FACSCalibur flow cytometer (BD Biosciences). Data analysis was performed using FlowJo (v.9.3, Treestar) by investigators blinded to microbiome composition.

For CD3 immunohistochemistry, OCT-cryopreserved tissues were sectioned and stained with anti-CD3 antibody (Vector Labs) and Hematoxylin (Fisher Scientific). One whole tissue section from each of the inner and outer aspects of the foreskin was analyzed per participant (median 6.10 mm^2^ tissue/participant). The epidermis and dermis (to a depth of 300 μm from the surface of the foreskin) were delineated using ImageJ (NIH) and CD3^+^ cells counted by an investigator blinded to both foreskin aspect (inner vs. outer) and microbiome composition. To convert proportions of T cell subsets obtained through flow cytometry to tissue densities, an overall T cell density was calculated for each man by averaging the density of CD3^+^ cells/mm^2^ in both the inner and outer aspects, including both epidermis and dermis, obtained through immunohistochemistry. This overall T cell density was multiplied by the percentage of CD3^+^ flow cytometry events falling into a subset of interest (i.e., the percentage of CD3^+^ events that were also CD4^+^CCR5^+^ was multiplied by the average density of CD3^+^ cells/mm^2^ tissue).

### Statistics

#### Association between penile microbiome composition and risk of seroconversion.

We first characterized the prevalence (prevalence of a taxon among participants), proportional abundance (proportional abundance of a taxon within an individual’s microbiome), and absolute abundance (log_10_ 16S RNA gene copies per swab) of penile bacterial genera, where prevalence was defined as the percentage of participants for whom we detected 10 or more sequences from the particular taxon. We next compared the overall microbiome composition in cases versus controls from the HIV seroconversion cohort by permutational analysis of variance. To further assess the relationship between overall microbiome composition and seroconversion risk, we ranked participants based on abundance of *Prevotella* to generate quartiles (PR Q1 to PR Q4, where PR Q denotes *Prevotella* quartile). We then compared the total bacterial density (log_10_ 16S RNA gene copies per swab) across *Prevotella* quartiles by analysis of variance and assessed the associations between HIV seroconversion and (a) *Prevotella* quartile assignment and (b) total bacterial density using logistic regression.

#### Definition of penile bacterial species associated with seroconversion.

Seroconversion-associated genera were defined as those that had a statistically significant (*P <* 0.05) OR in multivariate logistic regression of seroconversion on absolute abundance (adjusting for number of sexual partners, condom use, and age) and had a median absolute abundance above the first quartile (which excluded only taxa with proportional abundance <0.001). Using species-level data from each of these seroconversion-associated genera, as well as species that have been previously reported in the literature as being associated with seroconversion in either men or women (3, 4), we then identified seroconversion-associated species based on having a statistically significant (*P <* 0.05) OR in multivariate logistic regression of seroconversion on absolute abundance (adjusting for number of sexual partners, condom use, and age). This initial screening analysis was inclusive; we did not control for multiple comparisons (which would protect against false positives but increase the likelihood of false negatives), and we included additional species from genera that were not associated with seroconversion in the present analysis if they been found to be associated with seroconversion in previous publications. This inclusive approach protected against missing potentially important species-level associations (false negatives), while subsequent analyses assessing relationships with cellular markers and cytokines were employed to screen out false positives.

#### Assessment of immune associations.

From the seroconversion-associated species, final species associated with both subpreputial IL-8 level and foreskin CCR5^+^CD4^+^ T cell density were identified using Spearman correlations with the absolute abundance of each penile bacterial species. These final species were defined as the BASIC species. We assessed the specificity of immune response associated with BASIC species abundance by comparing 3 groups from the cellular immunology cohort, selected based on abundance of BASIC species, and 4 control taxa: 2 taxa of anaerobes reduced by male circumcision but not associated with seroconversion (*Negativicoccus spp*. and *Helcococcus spp.*) and 2 taxa increased by male circumcision (*Corynebacterium spp*. and *Staphylococcus spp.*). Groups were defined as follows: (a) group A, participants with a high abundance of BASIC species, defined as individuals within the top quartile of BASIC absolute abundance but not within the top quartile of absolute abundance of control taxa; (b) group B, participants with high abundance of control taxa, defined as individuals within the top quartile of control taxa absolute abundance but not within the top quartile of BASIC absolute abundance; and (c) group C, participants within the bottom quartile of BASIC species but not in the top quartile of control taxa (so as to generate mutually exclusive groups). We compared bacterial densities, IL-8 concentrations, and T cell densities among the 3 microbiome groups using the Wilcoxon rank-sum test. We further evaluated localization of foreskin T cells by comparing T cell density in the inner and outer foreskin epidermis and dermis of the 3 microbiome groups by immunohistochemistry using the Wilcoxon rank-sum test to compare T cell density between groups and the Wilcoxon signed-rank test to compare if difference between dermis and epidermis is significantly different from 0.

#### Assessment of bacterial co-occurrence.

To assess penile bacterial co-occurrence patterns, we utilized network analysis. We constructed the co-occurrence network using pairwise Spearman correlations based on absolute abundances and graphed the 6 BASIC species and 4 control taxa. Up to 10 most strongly and significantly (*P <* 0.01) correlated genera/species for the BASIC species and control taxa were also included. To assess the relationship between penile bacteria and immune parameters, we further annotated the penile bacterial networks with Spearman correlations between each taxon and subpreputial IL-8 levels and CCR5^+^ CD4^+^ T cell densities.

#### Statistical software.

All statistical analyses were performed using R (R Core Team, 2018; http://wwwR-project.org/), SAS (SAS Institute Inc., version 9.4), and StataSE (StataCorp, version 16). Graphs were prepared in R and Prism (GraphPad, version 8). Networks were spatialized in Gephi 0.9.2 using Force Atlas 2 (28).

### Study approval

All participants provided written informed consent before enrollment. Ethical approval for all studies was obtained from institutional review boards at Western University, the University of Toronto, the Uganda Virus Research Institute Scientific Ethics Committee (Entebbe, Uganda), and the Johns Hopkins University Bloomberg School of Public Health.

## Author contributions

JLP, LB, and RK performed immunology experiments. AGA provided statistical oversight. AART contributed to study design and data analysis. DEP, MA, KR, and CML performed microbiome analyses. RHG, GK, RMG, JS, JN, and JK oversaw participant enrollment and sample collection. All authors contributed to study design, scientific discussions, and manuscript preparation.

## Supplementary Material

Supplemental data

## Figures and Tables

**Table 1:**
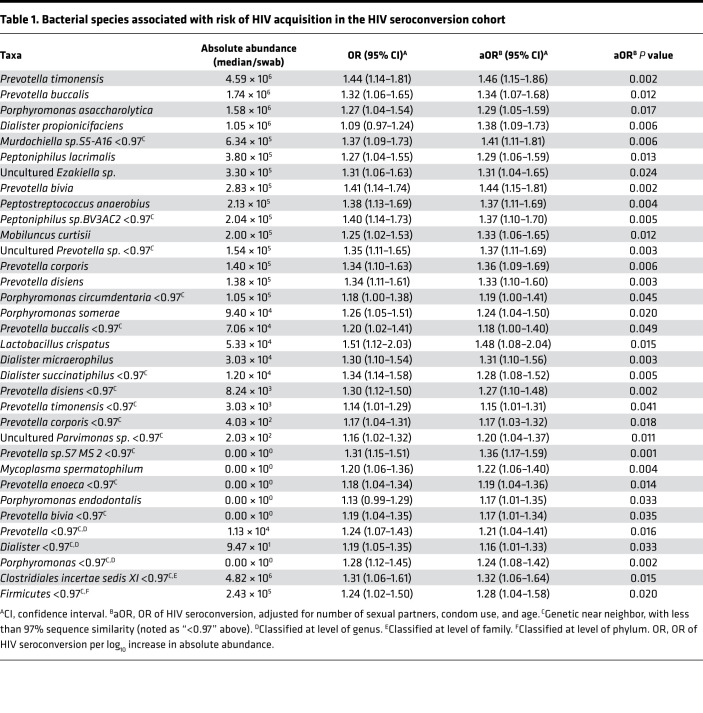
Bacterial species associated with risk of HIV acquisition in the HIV seroconversion cohort

**Table 2:**
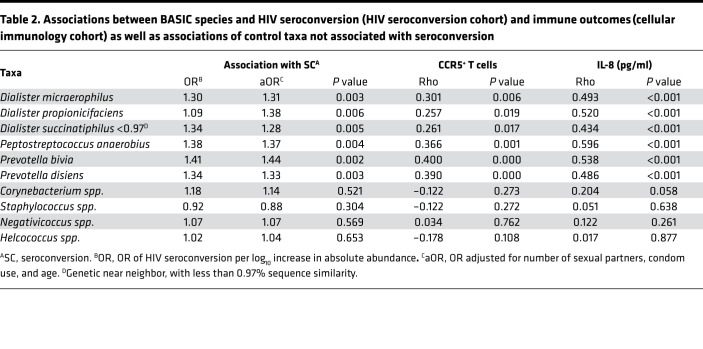
Associations between BASIC species and HIV seroconversion (HIV seroconversion cohort) and immune outcomes (cellular immunology cohort) as well as associations of control taxa not associated with seroconversion

## Version 1 04/22/2021

Electronic publication
