# Supplementary material for: Penile bacteria associated with HIV seroconversion, inflammation, and immune cells
Source: JCI Insight. 2021 Apr 22;6(8):e147363. doi: 10.1172/jci.insight.147363 (PMC8119186; doi:10.1172/jci.insight.147363)
Supplement: Supplemental data [file jciinsight-6-147363-s127.pdf]

Table S1.
Proportional abundance, and absolute abundance of penile bacteria from the 266 men included in the HIV Seroconversion Cohort. All men were uncircumcised.

	Proportional Abundance			Absolute Abundance (16S copies/swab)		
**Genus**	**Median**	**Q[#]1**	**Q[#]3**	**Median**	**Q[#]1**	**Q[#]3**
Prevotella	0.155	0.059	0.279	2.78E+07	9.78E+05	3.14E+08
Peptoniphilus	0.077	0.038	0.119	1.52E+07	1.66E+06	8.69E+07
Peptoniphilaceae<0.97[*†]	0.049	0.013	0.125	7.81E+06	2.97E+05	1.39E+08
Porphyromonas	0.032	0.010	0.067	4.47E+06	2.36E+05	9.88E+07
Finegoldia	0.029	0.005	0.104	3.37E+06	7.02E+05	2.07E+07
Corynebacterium	0.027	0.004	0.119	2.74E+06	7.12E+05	1.19E+07
Anaerococcus	0.025	0.008	0.061	4.38E+06	7.03E+05	2.10E+07
Dialister	0.018	0.002	0.045	1.76E+06	7.01E+04	3.40E+07
Lactobacillus	0.002	0.001	0.004	2.46E+05	3.33E+04	2.33E+06
Gardnerella	0.001	0.000	0.003	5.72E+04	9.27E+03	8.40E+05
Ezakiella	0.004	0.001	0.016	3.59E+05	2.59E+04	1.11E+07
Clostridiales incertae sedis XIII<0.97[*†]	0.003	0.000	0.010	3.80E+05	1.32E+04	1.10E+07
Mobiluncus	0.001	0.000	0.011	2.24E+05	1.18E+04	6.54E+06
Firmicutes<0.97[*‡]	0.005	0.001	0.016	4.55E+05	1.76E+04	1.55E+07
Murdochiella	0.004	0.001	0.015	6.34E+05	2.70E+04	1.10E+07
Campylobacter	0.004	0.000	0.013	4.19E+05	1.69E+04	1.25E+07
Negativicoccus	0.003	0.000	0.018	3.83E+05	2.54E+04	6.11E+06
Saccharofermentans<0.97[*]	0.001	0.000	0.009	9.49E+04	7.09E+03	5.02E+06
Peptostreptococcus	0.004	0.000	0.027	2.13E+05	1.69E+04	1.38E+07
Proteobacteria<0.97[*‡]	0.001	0.000	0.010	9.69E+04	7.26E+03	3.90E+06
Porphyromonas<0.97[*]	0.001	0.000	0.005	1.08E+05	4.03E+03	3.46E+06
Staphylococcus	0.000	0.000	0.004	8.00E+04	1.63E+04	3.13E+05
Clostridiales incertae sedis XI<0.97[*†]	0.001	0.000	0.003	1.04E+05	3.67E+03	2.42E+06
Escherichia/Shigella	0.000	0.000	0.002	3.52E+04	1.44E+04	1.32E+05
Pyramidobacter	0.000	0.000	0.003	2.85E+04	1.32E+03	1.19E+06

Peptococcus	0.001	0.000	0.002	1.17E+05	4.66E+03	2.49E+06
Streptococcus	0.000	0.000	0.002	2.59E+04	4.76E+03	2.32E+05
Jonquetella	0.000	0.000	0.001	1.60E+04	1.68E+03	3.01E+05
Actinomyces	0.000	0.000	0.001	6.25E+04	4.49E+03	5.74E+05
Bacteroidia<0.97[*§]	0.000	0.000	0.003	2.93E+04	1.75E+03	2.05E+06
Varibaculum	0.000	0.000	0.002	4.50E+04	3.14E+03	8.46E+05
Sneathia<0.97[*]	0.000	0.000	0.002	2.56E+04	1.96E+03	4.25E+05
Veillonella	0.000	0.000	0.002	3.19E+04	2.70E+03	4.58E+05
Murdochiella<0.97[*]	0.000	0.000	0.002	2.30E+04	1.48E+03	8.33E+05
Fusobacterium	0.000	0.000	0.002	2.96E+04	1.71E+03	5.29E+05
Prevotellaceae<0.97[*†]	0.000	0.000	0.002	1.97E+04	1.23E+03	7.41E+05
Atopobium	0.000	0.000	0.001	1.30E+04	8.36E+02	2.31E+05
Spirochaetes<0.97[*‡]	0.000	0.000	0.001	1.21E+04	4.03E+02	5.16E+05
Parvibacter<0.97[*]	0.000	0.000	0.000	1.28E+04	3.50E+02	2.21E+05
Bacteroidetes<0.97[*‡]	0.000	0.000	0.001	7.90E+03	4.00E+02	2.65E+05
Peptostreptococcaceae<0.97[*†]	0.000	0.000	0.001	2.55E+04	3.88E+02	4.84E+05
Olsenella	0.000	0.000	0.001	8.59E+03	0.00E+00	2.18E+05
Porphyromonadaceae<0.97[*†]	0.000	0.000	0.001	9.35E+03	0.00E+00	8.89E+05
Dietziaceae<0.97[*†]	0.000	0.000	0.001	8.35E+03	0.00E+00	1.43E+05
Moryella	0.000	0.000	0.001	1.46E+04	0.00E+00	7.18E+05
Olsenella<0.97[*]	0.000	0.000	0.001	1.65E+04	0.00E+00	3.07E+05
Clostridia<0.97[*§]	0.000	0.000	0.001	8.37E+03	0.00E+00	4.45E+05
Stomatobaculum<0.97[*]	0.000	0.000	0.000	4.55E+03	0.00E+00	2.74E+05
Acinetobacter	0.000	0.000	0.001	5.04E+03	0.00E+00	4.73E+04
Howardella<0.97[*]	0.000	0.000	0.000	1.83E+04	0.00E+00	3.59E+05
Facklamia	0.000	0.000	0.000	1.09E+04	0.00E+00	1.28E+05
Saccharibacteria incertae sedis	0.000	0.000	0.001	3.52E+03	0.00E+00	8.27E+04
Enterorhabdus<0.97[*]	0.000	0.000	0.000	2.94E+03	0.00E+00	1.16E+05
Parvimonas	0.000	0.000	0.001	3.54E+03	0.00E+00	2.38E+05
Sutterella	0.000	0.000	0.001	3.06E+03	0.00E+00	3.01E+05
Succiniclasticum<0.97[*]	0.000	0.000	0.000	2.23E+03	0.00E+00	1.73E+05

*Synergistetes<0.97**‡	0.000	0.000	0.001	3.60E+03	0.00E+00	3.73E+05
Micrococcus	0.000	0.000	0.000	2.30E+03	0.00E+00	1.75E+04
*Ruminococcaceae<0.97**†	0.000	0.000	0.000	3.23E+03	0.00E+00	1.57E+05
Arcanobacterium	0.000	0.000	0.000	2.92E+03	0.00E+00	1.80E+05
Sneathia	0.000	0.000	0.000	1.34E+03	0.00E+00	5.78E+04
*Sphingobacteriia<0.97**§	0.000	0.000	0.000	1.30E+03	0.00E+00	7.58E+04
*Megasphaera<0.97**	0.000	0.000	0.000	1.20E+03	0.00E+00	3.61E+04
*Candidatus Saccharibacteria<0.97**‡	0.000	0.000	0.000	1.95E+03	0.00E+00	1.21E+05
Mycoplasma	0.000	0.000	0.000	1.41E+03	0.00E+00	1.01E+05
*Barnesiella<0.97**	0.000	0.000	0.000	7.43E+02	0.00E+00	3.44E+05
*Eremococcus<0.97**	0.000	0.000	0.000	1.11E+03	0.00E+00	2.01E+04
Granulicatella	0.000	0.000	0.000	5.45E+02	0.00E+00	3.61E+04
Gemella	0.000	0.000	0.000	6.41E+02	0.00E+00	1.45E+04
*Eubacteriaceae<0.97**†	0.000	0.000	0.000	5.02E+02	0.00E+00	5.77E+04
*Dialister<0.97**	0.000	0.000	0.000	9.47E+01	0.00E+00	5.61E+04
*Oribacterium<0.97**	0.000	0.000	0.000	0.00E+00	0.00E+00	8.06E+04
Pseudomonas	0.000	0.000	0.000	0.00E+00	0.00E+00	8.43E+03
Aerococcus	0.000	0.000	0.000	0.00E+00	0.00E+00	1.17E+04
*Peptostreptococcaceae incertae sedis<0.97**	0.000	0.000	0.000	0.00E+00	0.00E+00	2.19E+04
*Shuttleworthia<0.97**	0.000	0.000	0.000	0.00E+00	0.00E+00	1.35E+04
*Dermabacter<0.97**	0.000	0.000	0.000	0.00E+00	0.00E+00	1.07E+04
*Lachnospiracea incertae sedis<0.97**	0.000	0.000	0.000	0.00E+00	0.00E+00	1.49E+04
*Flavonifractor<0.97**	0.000	0.000	0.000	0.00E+00	0.00E+00	2.69E+04
*Treponema<0.97**	0.000	0.000	0.000	0.00E+00	0.00E+00	1.02E+04
SR1 genera incertae sedis	0.000	0.000	0.000	0.00E+00	0.00E+00	1.57E+04
*Bacteroidales incertae sedis<0.97**†	0.000	0.000	0.000	0.00E+00	0.00E+00	1.39E+04
*Enterobacter<0.97**	0.000	0.000	0.000	0.00E+00	0.00E+00	2.18E+03
Helcococcus	0.000	0.000	0.000	0.00E+00	0.00E+00	3.16E+03
Neisseria	0.000	0.000	0.000	0.00E+00	0.00E+00	2.28E+03
Bacteroides	0.000	0.000	0.000	0.00E+00	0.00E+00	2.49E+03
Chryseobacterium	0.000	0.000	0.000	0.00E+00	0.00E+00	1.55E+03

Comamonas	0.000	0.000	0.000	0.00E+00	0.00E+00	9.95E+02
Treponema	0.000	0.000	0.000	0.00E+00	0.00E+00	2.64E+03
Eubacterium	0.000	0.000	0.000	0.00E+00	0.00E+00	7.70E+03
Haemophilus<0.97[*]	0.000	0.000	0.000	0.00E+00	0.00E+00	8.55E+02
Citrobacter<0.97[*]	0.000	0.000	0.000	0.00E+00	0.00E+00	3.83E+02
Megasphaera	0.000	0.000	0.000	0.00E+00	0.00E+00	7.21E+02
Desulfovibrio<0.97[*]	0.000	0.000	0.000	0.00E+00	0.00E+00	6.98E+02
Haemophilus	0.000	0.000	0.000	0.00E+00	0.00E+00	3.92E+02
Selenomonas<0.97[*]	0.000	0.000	0.000	0.00E+00	0.00E+00	2.74E+02
Aeromonas	0.000	0.000	0.000	0.00E+00	0.00E+00	0.00E+00
Alloprevotella	0.000	0.000	0.000	0.00E+00	0.00E+00	0.00E+00
Fretibacterium	0.000	0.000	0.000	0.00E+00	0.00E+00	0.00E+00
Klebsiella	0.000	0.000	0.000	0.00E+00	0.00E+00	0.00E+00
Morganella	0.000	0.000	0.000	0.00E+00	0.00E+00	0.00E+00
Tenericutes<0.97[*‡]	0.000	0.000	0.000	0.00E+00	0.00E+00	0.00E+00
Sphingobacterium	0.000	0.000	0.000	0.00E+00	0.00E+00	0.00E+00
Cytophagia<0.97[*§]	0.000	0.000	0.000	0.00E+00	0.00E+00	0.00E+00
Cloacibacillus<0.97[*]	0.000	0.000	0.000	0.00E+00	0.00E+00	0.00E+00
Lactobacillales<0.97[*¶]	0.000	0.000	0.000	0.00E+00	0.00E+00	0.00E+00
Proteiniphilum	0.000	0.000	0.000	0.00E+00	0.00E+00	0.00E+00

[*]Genetic near neighbour, with <0.97% sequence similarity
[†]Classified at the level of Family
[¶]Classified at the level of Order
[§]Classified at the level of Class
[‡]Classified at the level of Phylum
[#]Q, quartile

Table S2.
The 11 bacterial genera associated with increased risk for subsequent seroconversion in the HIV Seroconversion Cohort, as well as 6 genera previous reported to be associated with seroconversion *(2-4)*.

**Taxa**	**Proportional abundance**	**OR (95% CI[#])**	**aOR[§] (95% CI[#])**	**aOR p-value**
Prevotella	0.155	1.42 (1.11-1.81)	1.427 (1.11-1.84)	0.006
Peptoniphilaceae[†]	0.049	1.32 (1.05-1.64)	1.332 (1.06-1.68)	0.012
Porphyromonas	0.032	1.26 (1.01-1.58)	1.286 (1.02-1.63)	0.038
Dialister	0.018	1.39 (1.12-1.74)	1.39 (1.10-1.75)	0.007
Firmicutes<0.97[*‡]	0.005	1.27 (1.04-1.54)	1.315 (1.07-1.62)	0.010
Murdochiella	0.004	1.37 (1.09-1.73)	1.414 (1.11-1.81)	0.006
Peptostreptococcus	0.004	1.38 (1.13-1.69)	1.365 (1.11-1.69)	0.004
Ezakiella	0.004	1.31 (1.06-1.63)	1.306 (1.04-1.64)	0.023
Clostridiales incertae sedis XI<0.97[*†]	0.003	1.21 (1.01-1.44)	1.211 (1.01-1.45)	0.040
Mobiluncus	0.001	1.21 (1.00-1.48)	1.293 (1.04-1.61)	0.021
Porphyromonas<0.97[*]	0.001	1.26 (1.05-1.50)	1.252 (1.04-1.50)	0.017
Finegoldia (2)	0.029	1.44 (1.04-2.00)	1.374 (0.98-1.93)	0.068
Peptoniphilus (2)	0.077	1.39 (1.03-1.87)	1.378 (1.01-1.88)	0.042
Lactobacillus (3, 4)	0.002	1.29 (0.98-1.68)	1.256 (0.94-1.68)	0.124
Gardnerella (3, 4)	0.001	1.28 (1.00-1.64)	1.236 (0.95-1.61)	0.111
Mycoplasma (3, 4)	0.000	1.19 (1.05-1.35)	1.201 (1.05-1.37)	0.007
Parvimonas (3, 4)	0.000	1.10 (0.97-1.25)	1.100 (0.96-1.26)	0.177

[*]Genetic near neighbour, with <0.97% sequence similarity
[†]Classified at the level of Family
[‡]Classified at the level of Phylum
[**]OR, odds ratio of HIV seroconversion per $\log_{10}$ increase in absolute abundance
[§]aOR, adjusted odds ratio of HIV seroconversion
[#]CI, confidence interval

Table S3.
Penile bacterial species belonging to the 14 genera associated with increased risk for subsequent seroconversion HIV Seroconversion Cohort.

Genus	Species	Absolute abundance	OR (95% CI[#])	aOR[§] (95% CI[#])	p-value
Clostridiales incertae sedis XI<0.97[*‡]		4824405	1.31 (1.06-1.61)	1.32 (1.06-1.64)	0.015
Dialister	_Dialister micraerophilus_	30312	1.30 (1.10-1.54)	1.31 (1.10-1.56)	<0.01
Dialister	_Dialister propionicifaciens_	1053429	1.37 (1.10-1.70)	1.38 (1.09-1.73)	<0.01
Dialister	_Dialister propionicifaciens<0.97_[*]	2553	1.09 (0.97-1.24)	1.09 (0.96-1.24)	0.183
Dialister	_Dialister succinatiphilus<0.97_[*]	11963	1.34 (1.14-1.58)	1.28 (1.08-1.52)	<0.01
Dialister	_Dialister<0.97_[*†]	95	1.19 (1.05-1.35)	1.16 (1.01-1.33)	0.033
Ezakiella	uncultured _Ezakiella sp._	329873	1.31 (1.06-1.63)	1.31 (1.04-1.65)	0.024
Finegoldia	_Finegoldia magna_	3370916	1.44 (1.04-2.00)	1.37 (0.98-1.93)	0.068
Firmicutes<0.97[*¶]		243166	1.24 (1.02-1.50)	1.28 (1.04-1.58)	0.020
Gardnerella	_Gardnerella vaginalis_	57206	1.28 (1.00-1.64)	1.24 (0.95-1.61)	0.111
Lactobacillus	_Lactobacillus crispatus_	53342	1.51 (1.12-2.03)	1.48 (1.08-2.04)	0.015
Lactobacillus	_Lactobacillus iners_	107051	1.25 (0.96-1.62)	1.20 (0.91-1.59)	0.197
Lactobacillus	_Lactobacillus reuteri<0.97_[*]	0	1.05 (0.91-1.21)	1.11 (0.95-1.30)	0.181
Mobiluncus	_Mobiluncus curtisii_	199773	1.25 (1.02-1.53)	1.33 (1.06-1.65)	0.012
Mobiluncus	_Mobiluncus curtisii<0.97_[*]	0	1.08 (0.94-1.24)	1.11 (0.95-1.28)	0.194
Mobiluncus	uncultured _Mobiluncus sp.<0.97_[*]	0	1.04 (0.92-1.18)	1.09 (0.95-1.25)	0.222
Murdochiella	_Murdochiella<0.97_[*†]	1262	1.11 (0.97-1.27)	1.11 (0.97-1.28)	0.139
Murdochiella	_Murdochiella sp.S5-A16<0.97_[*]	634098	1.37 (1.09-1.73)	1.41 (1.11-1.81)	<0.01
Mycoplasma	_Mycoplasma hominis_	0	1.01 (0.87-1.19)	0.96 (0.81-1.15)	0.651
Mycoplasma	_Mycoplasma orale_	0	1.19 (0.94-1.50)	1.20 (0.93-1.56)	0.161
Mycoplasma	_Mycoplasma spermatophilum_	0	1.20 (1.06-1.36)	1.22 (1.06-1.40)	<0.01
Parvimonas	uncultured _Parvimonas sp.<0.97_[*]	203	1.16 (1.02-1.32)	1.20 (1.04-1.37)	0.011
Peptoniphilaceae[‡]		29591	1.13 (0.98-1.31)	1.15 (0.99-1.34)	0.067
Peptoniphilaceae<0.97[*‡]		29591	1.13 (0.98-1.31)	1.15 (0.99-1.34)	0.067
Peptoniphilus	_Peptoniphilus grossensis ph5<0.97_[*]	24294	1.14 (0.97-1.34)	1.14 (0.96-1.34)	0.127
Peptoniphilus	_Peptoniphilus lacrimalis_	379916	1.27 (1.04-1.55)	1.29 (1.06-1.59)	0.013

Peptoniphilus	Peptoniphilus sp BV3AC2<0.97[*]	203676	1.40 (1.14-1.73)	1.37 (1.10-1.70)	<0.01
Peptoniphilus	Peptoniphilus timonensis JC401	3280	1.11 (0.98-1.26)	1.10 (0.97-1.26)	0.147
Peptoniphilus	Peptoniphilus<0.97[*†]	176994	1.14 (0.93-1.38)	1.12 (0.91-1.37)	0.293
Peptostreptococcus	Peptostreptococcus anaerobius	212705	1.38 (1.13-1.69)	1.37 (1.11-1.69)	<0.01
Porphyromonas	Porphyromonas asaccharolytica	1583845	1.27 (1.04-1.54)	1.29 (1.05-1.59)	0.017
Porphyromonas	Porphyromonas bennonis	1323164	1.19 (0.94-1.51)	1.22 (0.95-1.58)	0.123
Porphyromonas	Porphyromonas circumdentaria<0.97[*]	105176	1.18 (1.00-1.38)	1.19 (1.00-1.41)	0.045
Porphyromonas	Porphyromonas endodontalis	0	1.13 (0.99-1.29)	1.17 (1.01-1.35)	0.033
Porphyromonas	Porphyromonas somerae	93971	1.26 (1.05-1.51)	1.24 (1.04-1.50)	0.020
Porphyromonas	Porphyromonas sp.2033b<0.97[*]	0	0.91 (0.77-1.08)	0.89 (0.74-1.07)	0.215
Porphyromonas	Porphyromonas<0.97[*†]	0	1.28 (1.12-1.45)	1.24 (1.08-1.42)	<0.01
Prevotella	Prevotella<0.97[*†]	11253	1.24 (1.07-1.43)	1.21 (1.04-1.41)	0.016
Prevotella	Prevotella amnii	0	1.12 (0.98-1.27)	1.07 (0.93-1.24)	0.336
Prevotella	Prevotella bergensis	43268	1.10 (0.95-1.28)	1.11 (0.95-1.31)	0.189
Prevotella	Prevotella bivia	282609	1.41 (1.14-1.74)	1.44 (1.15-1.81)	<0.01
Prevotella	Prevotella bivia<0.97[*]	0	1.19 (1.04-1.35)	1.17 (1.01-1.34)	0.035
Prevotella	Prevotella buccalis	1735590	1.32 (1.06-1.65)	1.34 (1.07-1.68)	0.012
Prevotella	Prevotella buccalis<0.97[*]	70563	1.20 (1.02-1.41)	1.18 (1.00-1.40)	0.049
Prevotella	Prevotella corporis	140115	1.34 (1.10-1.63)	1.36 (1.09-1.69)	<0.01
Prevotella	Prevotella corporis<0.97[*]	403	1.17 (1.04-1.31)	1.17 (1.03-1.32)	0.018
Prevotella	Prevotella disiens	138477	1.34 (1.11-1.61)	1.32 (1.10-1.60)	<0.01
Prevotella	Prevotella disiens<0.97[*]	8238	1.30 (1.12-1.50)	1.27 (1.10-1.48)	<0.01
Prevotella	Prevotella enoeca<0.97[*]	0	1.18 (1.04-1.34)	1.19 (1.04-1.36)	0.014
Prevotella	Prevotella intermedia	0	1.16 (1.02-1.33)	1.13 (0.99-1.30)	0.076
Prevotella	Prevotella melaninogenica	0	1.15 (1.00-1.32)	1.15 (0.99-1.34)	0.073
Prevotella	Prevotella sp.S7 MS 2<0.97[*]	0	1.31 (1.15-1.51)	1.36 (1.17-1.59)	<0.01
Prevotella	Prevotella sp.oral clone DO045	4065	1.08 (0.96-1.22)	1.12 (0.98-1.28)	0.088
Prevotella	Prevotella timonensis	4590384	1.44 (1.14-1.81)	1.46 (1.15-1.86)	<0.01
Prevotella	Prevotella timonensis<0.97[*]	3034	1.14 (1.01-1.29)	1.15 (1.01-1.31)	0.041
Prevotella	uncultured Prevotella sp<0.97[*]	153686	1.35 (1.11-1.65)	1.37 (1.11-1.69)	<0.01

*Genetic near neighbour, with <0.97% sequence similarity
†Classified at the level of Genus
‡Classified at the level of Family
¶Classified at the level of Phylum
**OR, odds ratio of HIV seroconversion per log_{10} increase in absolute abundance §aOR, adjusted odds ratio of HIV seroconversion
#CI, confidence interval

Table S4.
Associations of the 34 seroconversion-associated penile bacterial species— and four control taxa— with foreskin immune parameters in the two cohorts included in this study.

		HIV Seroconversion Cohort				Mucosal Immunology Cohort							
		IL-8 (pg/ml)		α–defensins (pg/ml)		IL-8 (pg/ml)		α-defensins (pg/ml)		CCR5+CD4+ (T cells/mm^2)		Th17 (cells/mm^2)	
**Taxa**	**Association with SC[§]**	**Rho**	**p-value**	**Rho**	**p-value**	**Rho**	**p-value**	**Rho**	**p-value**	**Rho**	**p-value**	**Rho**	**p-value**
Clostridiales incertae sedis XI<0.97[*¶]	Associated	0.445	**<0.001**	0.421	**<0.001**	0.366	**<0.001**	0.368	**0.003**	0.139	0.211	0.204	0.066
Dialister micraerophilus	Associated	0.590	**<0.001**	0.408	**<0.001**	0.493	**<0.001**	0.274	**0.031**	0.301	**0.006**	0.334	**0.002**
Dialister propionicifaciens	Associated	0.510	**<0.001**	0.428	**<0.001**	0.520	**<0.001**	0.398	**0.001**	0.257	**0.019**	0.334	**0.002**
Dialister succinatiphilus<0.97[*]	Associated	0.443	**<0.001**	0.377	**<0.001**	0.434	**<0.001**	0.354	**0.005**	0.261	**0.017**	0.266	**0.016**
Dialister<0.97[*†]	Associated	0.495	**<0.001**	0.432	**<0.001**	0.289	**0.008**	0.143	0.267	-0.006	0.958	0.010	0.930
Uncultured *Ezakiella sp.*	Associated	0.343	**<0.001**	0.342	**<0.001**	0.357	**<0.001**	0.336	**0.008**	0.183	0.098	0.176	0.114
Firmicutes<0.97[*‡]	Associated	0.406	**<0.001**	0.443	**<0.001**	0.349	**0.001**	0.394	**0.002**	0.105	0.345	0.125	0.264
Lactobacillus crispatus	Associated	0.383	**<0.001**	0.379	**<0.001**	0.253	**0.020**	0.293	**0.021**	0.078	0.482	-0.010	0.931
Mobiluncus curtisii	Associated	0.393	**<0.001**	0.418	**<0.001**	0.390	**<0.001**	0.398	**0.001**	0.159	0.151	0.159	0.154
Murdochiella sp.S5-A16<0.97[*]	Associated	0.335	**<0.001**	0.344	**<0.001**	0.368	**<0.001**	0.314	**0.013**	0.118	0.288	0.164	0.140
Mycoplasma spermatophilum	Associated	0.230	**0.002**	0.126	0.230	0.247	**0.024**	0.211	0.100	0.042	0.708	-0.041	0.714
Uncultured *Parvimonas sp.<0.97*[*]	Associated	0.491	**<0.001**	0.317	**0.002**	0.199	0.069	0.152	0.237	0.187	0.090	0.210	0.059
Peptoniphilus lacrimalis	Associated	0.443	**<0.001**	0.489	**<0.001**	0.432	**<0.001**	0.381	**0.002**	0.128	0.250	0.227	**0.041**
Peptoniphilus sp.BV3AC2<0.97[*]	Associated	0.368	**<0.001**	0.437	**<0.001**	0.399	**<0.001**	0.386	**0.002**	0.165	0.135	0.167	0.133
Peptostreptococcus anaerobius	Associated	0.587	**<0.001**	0.394	**<0.001**	0.596	**<0.001**	0.407	**0.001**	0.366	**0.001**	0.406	**0.000**
Porphyromonas asaccharolytica	Associated	0.455	**<0.001**	0.434	**<0.001**	0.358	**<0.001**	0.371	**0.003**	0.140	0.209	0.198	0.075
Porphyromonas circumdentaria<0.97[*]	Associated	0.439	**<0.001**	0.440	**<0.001**	0.385	**<0.001**	0.377	**0.003**	0.118	0.287	0.185	0.096

Porphyromonas endodontalis	Associated	0.174	**0.017**	0.210	**0.043**	0.151	0.170	0.103	0.425	-0.323	**0.003**	-0.280	**0.011**
Porphyromonas somerae	Associated	0.439	**<0.001**	0.443	**<0.001**	0.314	**0.004**	0.377	**0.003**	0.050	0.653	0.095	0.398
Porphyromonas<0.97[*†]	Associated	0.333	**<0.001**	0.282	**0.006**	0.341	**0.002**	0.362	**0.004**	0.128	0.248	0.113	0.313
Prevotella bivia	Associated	0.564	**<0.001**	0.384	**<0.001**	0.538	**<0.001**	0.356	**0.005**	0.400	**0.000**	0.435	**0.000**
Prevotella bivia<0.97[*]	Associated	0.412	**<0.001**	0.358	**<0.001**	0.353	**0.001**	0.387	**0.002**	0.175	0.113	0.189	0.089
Prevotella buccalis	Associated	0.335	**<0.001**	0.379	**<0.001**	0.379	**<0.001**	0.386	**0.002**	0.144	0.194	0.224	**0.043**
Prevotella buccalis<0.97[*]	Associated	0.365	**<0.001**	0.399	**<0.001**	0.279	**0.010**	0.255	**0.045**	0.071	0.524	0.141	0.206
Prevotella corporis	Associated	0.342	**<0.001**	0.255	**0.014**	0.400	**<0.001**	0.350	**0.005**	0.128	0.250	0.232	**0.036**
Prevotella corporis<0.97[*]	Associated	0.258	**<0.001**	0.352	**<0.001**	0.245	**0.025**	0.174	0.176	-0.030	0.790	0.162	0.146
Prevotella disiens	Associated	0.520	**<0.001**	0.396	**<0.001**	0.486	**<0.001**	0.342	**0.006**	0.390	**0.000**	0.420	**0.000**
Prevotella disiens<0.97[*]	Associated	0.507	**<0.001**	0.394	**<0.001**	0.379	**<0.001**	0.341	**0.007**	0.153	0.167	0.122	0.276
Prevotella enoeca<0.97[*]	Associated	0.176	**0.015**	0.282	**0.006**	0.273	**0.012**	0.100	0.441	0.023	0.834	0.108	0.333
Prevotella sp.S7 MS 2<0.97[*]	Associated	0.221	**0.001**	-0.036	0.695	0.435	**<0.001**	0.229	0.074	0.126	0.258	0.125	0.264
Prevotella timonensis	Associated	0.427	**<0.001**	0.397	**<0.001**	0.436	**<0.001**	0.391	**0.002**	0.167	0.131	0.249	**0.024**
Prevotella timonensis<0.97[*]	Associated	0.406	**<0.001**	0.388	**<0.001**	0.358	**<0.001**	0.272	**0.032**	0.047	0.674	0.105	0.349
Uncultured *Prevotella sp.<0.97*[*]	Associated	0.405	**<0.001**	0.378	**<0.001**	0.369	**<0.001**	0.467	**<0.001**	0.154	0.164	0.189	0.089
Prevotella<0.97[*†]	Associated	0.316	**<0.001**	0.362	**<0.001**	0.305	**0.005**	0.267	**0.036**	0.085	0.445	0.086	0.440
Corynebacterium spp.	Control	0.209	**0.004**	0.202	0.053	0.204	0.058	0.086	0.497	-0.122	0.273	-0.060	0.591
Staphylococcus spp.	Control	0.191	**0.009**	0.094	0.371	0.051	0.638	-0.058	0.649	-0.122	0.272	-0.079	0.479
Negativicoccus spp.	Control	0.163	**0.025**	0.196	0.059	0.122	0.261	0.167	0.184	0.034	0.762	0.101	0.367
Helcococcus spp.	Control	0.044	0.548	0.001	0.989	0.017	0.877	-0.028	0.828	-0.178	0.108	-0.130	0.246

[*]Genetic near neighbour, with <0.97% sequence similarity
[†]Classified at the level of Genus
[¶]Classified at the level of Family
[‡]Classified at the level of Phylum
[§]SC, seroconversion

Table S5.
Foreskin immune cell population densities in the Cellular Immunology Cohort, with high BASIC species abundance (Group A), high control taxa abundance (Group B), or low BASIC species abundance (Group C).

**Immune Cell Density** (median cells/mm^2)	**Group A**	**Group B**	**Group C**	**p-values**		
				A vs B	A vs. C	B vs C
T cells (CD3+)	162.7	35.9	22.4	**0.011**	**0.001**	0.311
Double Negative	13.1	4.2	2.5	**0.018**	**0.005**	0.500
CD8+	44.1	14.6	7.0	**0.009**	**0.003**	0.253
CD4+	86.0	15.7	9.7	**0.006**	**0.001**	0.176
CCR5+	30.0	6.2	4.0	**0.018**	**0.002**	0.138
Th17	6.1	1.2	0.6	**0.012**	**0.002**	0.253
Th22	0.7	0.1	0.1	**0.012**	**0.002**	0.832

Table S6.
Foreskin immune cell population proportional abundance in the Cellular Immunology Cohort, with high BASIC species abundance (Group A), high control taxa abundance (Group B), or low BASIC species abundance (Group C).

**Immune Cell Proportional Abundance** (median % of parent)	**Group A**	**Group B**	**Group C**	**p-values**		
				A vs B	A vs. C	B vs C
T cells (CD3+)	-	-	-			
Double Negative	9.7	9.2	12.7	0.453	0.204	0.090
CD8+	33.5	31.4	35.6	0.908	0.066	0.169
CD4+	53.5	52.7	48.0	0.773	**0.047**	0.103
CCR5+	57.5	57.4	49.1	0.387	0.472	0.672
Th17	6.7	6.5	6.7	0.689	0.897	0.627
Th22	1.0	0.7	1.0	0.196	0.890	0.880
